# Inefficiency of Rapid Urease Test for Confirmation of *Helicobacter pylori*

**DOI:** 10.4103/1319-3767.74441

**Published:** 2011

**Authors:** Amin Talebi Bezmin Abadi, Tarang Taghvaei, Lutz Wolfram

**Affiliations:** Department of Bacteriology, School of Medical Sciences, Tarbiat Modares University, Tehran, Iran; 1 Department of Internal Medicine, Faculty of Medicine, Mazandaran University of Medical Sciences, Sari, Iran; 2 Department of Internal Medicine, Division of Gastroenterology, University Hospital Zurich, Zurich, Switzerland. E-mail: amin.talebi@gmail.com

Sir,

Foroutan *et al*.[1] published an article entitled “Accuracy of rapid urease test in diagnosing *Helicobacter pylori* infection in patients using NSAIDs” in the previous volume of *Saudi Journal of Gastroenterology*. They have investigated the accuracy of rapid urease test (RUT) in *Helicobacter pylori* strains during the gastroscopy process. In response to their article, we present our contradictory evidences about the efficacy of RUT test as the confirmation of *H. pylori* isolates in biopsy specimen. Detection of *H. pylori* in clinical samples is an important subject for clinicians for further management of this rogue microorganism. Routinely, diagnosis tests of *H. pylori* are divided into two major groups:[2,3] invasive and noninvasive tests. At this point, we discuss RUT as one of the commonly used noninvasive test, which was applied by Foroutan *et al*.[1] It was recommended[2,3] that the presence of *H. pylori* should be confirmed by using at least any two of thee following methods: RUT, histology, UBT (Urea Breath Test), specific PCR assay, and bacterial culture. Nevertheless, they reported *H. pylori*-positive strains in their study, while no culture or specific PCR assay were performed in their examination.[1] Our data, supported by other reports from Iran,[4] demonstrate that most of the biopsy specimens from the antrum harbor *H. pylori*, while their RUT was primarily reported as being negative. Foroutan *et al*.[1] concluded that the use of NSAID does not affect the RUT test result. In our data[4,5] we observed that 30% of RUT-negative patients revealed *H. pylori* strains after culture and specific gene PCR assay.[2] In other words, we showed that of 340 consecutive patients referred for gastroscopy in our clinic, 95 (27%) were RUT negative.[4,5] This important finding, in contrast to Foroutan *et al*.[1] study, showed that RUT test does not show a high sensitivity if we perform in a condition with large sample size. In conclusion, we strongly recommended that physicians do not rely on RUT as a test of high sensitivity for test for determination of *H. pylori* infection.
